# Measurement of chest wall motion using a motion capture system with the one-pitch phase analysis method

**DOI:** 10.1038/s41598-021-01033-8

**Published:** 2021-11-02

**Authors:** Hiroyuki Tamiya, Akihisa Mitani, Hideaki Isago, Taro Ishimori, Minako Saito, Taisuke Jo, Goh Tanaka, Shintaro Yanagimoto, Takahide Nagase

**Affiliations:** 1 The Department of Respiratory Medicine, The University of Tokyo Hospital, 7-3-1, Hongo, Bunkyo-ku, Tokyo, 113-8655 Japan; 2 Health Service Center, The University of Tokyo, 7-3-1 Hongo, Bunkyo-ku, Tokyo, 113-8655 Japan; 3 The Department of Clinical Laboratory, The University of Tokyo Hospital, 7-3-1, Hongo, Bunkyo-ku, Tokyo, 113-8655 Japan; 4 The Division for Health Service Promotion, The University of Tokyo, 7-3-1, Hongo, Bunkyo-ku, Tokyo, 113-8655 Japan

**Keywords:** Respiratory tract diseases, Respiration

## Abstract

Spirometry is a standard method for assessing lung function. However, its use is challenging in some patients, and it has limitations such as risk of infection and inability to assess regional chest wall motion. A three-dimensional motion capture system using the one-pitch phase analysis (MCO) method can facilitate high precision measurement of moving objects in real-time in a non-contacting manner. In this study, the MCO method was applied to examine thoraco-abdominal (TA) wall motion for assessing pulmonary function. We recruited 48 male participants, and all underwent spirometry and chest wall motion measurement with the MCO method. A significant positive correlation was observed between the vital capacity (Spearman’s *ρ* = 0.68, *p* < 0.0001), forced vital capacity (Spearman’s *ρ* = 0.62, *p* < 0.0001), and tidal volume (Spearman’s *ρ* = 0.61, *p* < 0.0001) of spirometry and the counterpart parameters of MCO method. Moreover, the MCO method could detect regional rib cage and abdomen compartment contributions and could assess TA asynchrony, indicating almost complete synchronous movement (phase angle for each compartment: − 5.05° to 3.86°). These findings suggest that this technique could examine chest wall motion, and may be effective in analyzing chest wall volume changes and pulmonary function.

## Introduction

Spirometry is the standard method for assessing pulmonary function. Moreover, it is utilized in different situations including screening of general respiratory health; diagnosis and validation of severity of respiratory diseases such as asthma, chronic obstructive pulmonary disease (COPD), and interstitial lung disease; preoperative risk assessment; and monitoring of therapeutic intervention^1,2^. However, this technique has several limitations. First, participants who cannot follow instructions such as infants, patients with dementia and confusional state, and those in critical condition are not qualified because spirometry requires effort and cooperation. Second, it may cause infection through microorganism transfer via mouthpiece^3,4^. Third, it cannot detect compartmental movement of the chest wall. Thus, thoraco-abdominal asynchrony (TAA), non-parallel motion, or even opposing movement of the rib cage (RC) and the abdomen (ABD) during respiration, which may occur after chest surgery and in asthma, COPD, and neuromuscular disorders, cannot be identified^5^.

To address these problems, alternative methods including respiratory magnetometry^6^, respiratory inductive plethysmography^7^ and optoelectronic plethysmography (OEP) have been developed^8^. Most techniques are based on motion capture systems, which can analyze the three-dimensional movement of markers attached on the thoraco-abdominal (TA) surface during breathing. These methods can facilitate measurement of noninvasive volume changes in each TA wall compartment, and their efficacy has been well validated in healthy participants and in individuals with respiratory and musculoskeletal diseases. Nevertheless, these techniques are complex and expensive, and they should be performed by well-trained technicians. Hence, they are rarely applied in routine clinical practice^9^. Although time-of-flight depth camera sensor also can measure thoracic wall motion^10–13^, some of these methods apply marker attachment or use multiple cameras. Yu et al. and Sharp et al. described thoracic volume analysis using Kinect with a single camera and a non-contact manner^11,12^. However, structured pattern projection methods used in Kinect rely on the accuracy of the pixel unit. It is less accurate than phase analysis methods, which can analyze at subpixel resolution^14^. Therefore, novel methodologies for measuring TA wall movement which establish both accuracy and simplicity are required.

Morimoto et al. developed the one-pitch phase analysis method using moiré topography. This technique facilitates real-time, three-dimensional shape measurement of objects moving at high speed in a non-contacting manner^15^. Moreover, it can analyze the phase of grating images using information from one pitch of the projected grating with a single-shot image. The moiré topography does not require marker attachment and highly trained professionals. Hence, a larger number of participants can be evaluated within a short period. Moiré topography is reproducible, and has been applied to the detection of corporal asymmetries including scoliosis^16–18^. The usefulness of the one-pitch phase analysis method was evaluated in measuring the shape of moving human fingers and hands^14^. However, its use in measuring TA wall motion during respiration and its relationship with pulmonary function have not been fully elucidated. The current study aimed to investigate the feasibility of real-time measurement of chest wall volume changes using a motion capture system with the one-pitch phase analysis (MCO) method in healthy participants.

## Methods

### Participants

In total, 48 male students who underwent a health check-up in the Health Service Center, The University of Tokyo, on March 30, 2019, were included in this study. Data on smoking habits and previous medical history of asthma were obtained. A written informed consent was obtained before enrollment from all participants. The study protocol was approved by the institutional review board of The University of Tokyo Health Service Center (approval number: 17-249), and all research was performed in accordance with the Declaration of Helsinki.

### Procedures

All participants enrolled in this study initially underwent spirometry; then, chest wall motion analysis was performed using the MCO method after an interval of ≥ 5 min. Arterial oxygen saturation and pulse rate were assessed using a pulse oximeter (PULSOX 300i, Konica Minolta, Tokyo, Japan). In addition, height, weight, and chest circumference at the end of inspiration and expiration were evaluated. Body mass index (BMI) was calculated as body weight in kilograms divided by height in meters squared.

### Pulmonary function test

Spirometry was performed using CHESTGRAPH HI-301U (Chest M.I., Inc., Tokyo, Japan), and all measurements were performed according to the American Thoracic Society/European Respiratory Society guidelines^2,19^. Measurements were obtained while participants were sitting. At least three tests were conducted until satisfactory flow-volume curves were obtained. The following variables were measured: vital capacity (VC), forced vital capacity (FVC), expiratory forced volume within 1 s (FEV1), tidal volume (TV), peak expiratory flow (PEF), forced expiratory flow between 25 and 75% of FVC (FEF25–75), forced expiratory flow at 50% of FVC (FEF50), and forced expiratory flow at 25% of FVC (FEF75). The results were assessed by a respiratory physician, and the highest FEV1 and FVC values were selected. The predicted values for each variable were derived using the Japanese criteria^20^.

### Chest wall motion analysis

#### Setting of the equipment

**Figure 1:**
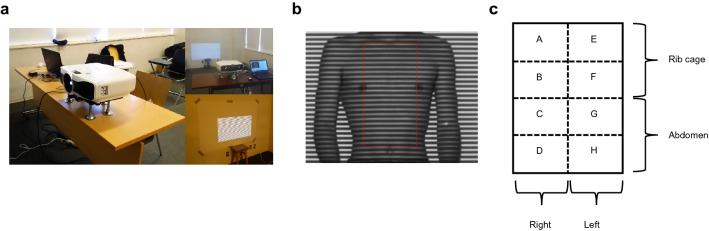
The structure of MCO and an example image by MCO method (participant No.1). **(a)** The system consists of a projector (upper part) and a camera (below the projector). A grating pattern is projected to the wall (3 m distance). Participants are asked to sit upright as far back as possible in a chair positioned close to the wall. **(b)** A grating pattern is projected onto the body surface of a subject. The red rectangle shows the surface area for analysis. **(c)** Regional definition of rib cage and abdomen for the surface area. *MCO* motion capture system using one-pitch phase analysis.

TA wall displacement signals and amount of displacement over a certain period were analyzed using a real-time three-dimensional shape measurement system with the MCO method (4D Sensor Inc., Wakayama, Japan; Fig. 1a). The grating pattern was projected onto the body surface, and the TA wall motion was recorded using a digital camera. This system extracts only one pitch of a grating from one grating image and analyzes its phase, and it can examine all pixel points of the image simultaneously. Therefore, phases can be analyzed at high speed (in real-time) and accuracy (20 frames per second). The detailed mechanism of the MCO method was described previously^14,15^. The participants were bare-chested, and they sat upright as far back as possible in a 45-cm-tall chair positioned close to the wall. We chose seated posture for MCO method analysis because spirometry was also conducted in the same positioning. A lustreless laminate film (product number: 50700022, FUJITEX Co., Tokyo, Japan) was attached on the wall. Images captured using a camera were translated into a virtual surface representing each participant’s chest wall under the following conditions: the projector, EH-TW8200W (Seiko Epson Corporation, Japan); digital camera, STC-MBS241U3V (Omron Sentech); sensor, IMX174 (Sony); lens, f = 8 mm; software used to analyze the captured image, 4D sensors’ own making software; the distance between the optical system and the wall behind the participant, 2.6 m; frame rate, 20 frames per second; resolution, 640 × 480 pixels; and images taken per participant, 6000. Window blinds were closed, and room lights were turned off because ambient light would interfere with the projected light from the optical system. The surface area for measuring chest wall motion was defined as follows: from the clavicle to the umbilicus in a vertical direction and from the right to the left nipple in a horizontal direction. The upper half of the area was defined as RC and the lower one as ABD. The RC area was divided into two parts: the upper RC (area A + E) and lower RC (area B + F), as the rib cage has been considered as a two-compartment system^8,21^. These anatomical landmarks were visually confirmed by researchers and surface area was fitted manually for each participant. The height of the device’s camera was adjusted such that the surface area was within the projected grating. A zero-point correction value was set on the wall behind the participant. The distance between optical system and the participants’ body surface (z) was expressed in mm. The 2-dimension image of the chest wall displacement was originally expressed in pixel (pixel width, x; pixel height, y). The actual width and height of the pixel in mm were obtained by measuring the size of the reference scale and calculating the magnification before the analysis. Then, the volume of the chest wall was measured by multiplying the width (x), height (y) of the pixel and the distance (z) together. Therefore, displacement of the body surface was considered as chest wall volume changes. In the MCO method, the pitch length of the grating on the camera imaging plane is always constant. Thus, it enables extracting the brightness data for one pitch at equal intervals and to carry out the phase analysis accurately, regardless of the distance from the optical system and the participant. Consequently, it does not require filter processing for aliasing removal^14^. The images of the chest wall were processed in real-time. Each participant underwent a single assessment under tidal breathing mode for 1 min, slow VC mode (inspire slowly and maximally, then exhale slowly and maximally) and three assessments of forced breathing mode for 1 min (inspire rapidly and maximally, then exhale forcibly and maximally). During the procedure, participants were instructed to keep still.

#### Synchronous movement analysis

The chest wall model of Konno and Mead^22^ was applied to analyze TAA and hemithoracic asynchrony (HTA). The wall displacement signals of the two compartments were plotted against each other (Konno–Mead diagram). The opening angle of the figure indicated the level of asynchrony between the compartments^23^. The two regions were considered synchronous when they moved closely to one another (indicated by an elliptical circle or almost a straight line: Supplementary Fig. S1a). The two regions were considered asynchronous when the movement of one region was delayed or led to another (indicated by a nearly perfect circle: Supplementary Fig. S1b). If two regions moved paradoxically, the circle was positioned downward to the right (Supplementary Fig. S1c). Asynchrony level (phase angle) was calculated as arcsin (*m*/*s*) based on the Konno–Mead diagram, where *m* was the width of the loop at 50% of *y*-axis (i.e., RC) displacement and *s* was the range of *x*-axis (e.g., ABD) displacement (Supplementary Figure S1d). Asynchrony was presented as degrees: 0° represented a perfect synchrony between the two compartments and 180° total asynchrony. A positive angle indicated that the motion of the area drawn on the y-axis was followed by the motion of the area drawn on the x-axis, and a negative angle suggested an opposite situation, as previously described^23–27^. For asynchronous analysis, the inspiratory paradox time (IPT) and expiratory paradox time (EPT) of the upper RC, lower RC, and ABD were also calculated. These parameters were defined as the fraction of time relative to the total chest wall displacement signal (expressed as percentage), in which the compartmental signals decreased during inspiration (IPT) and increased during expiration (EPT)^25,26,28,29^.

### Statistical analysis

Physical examination and spirometry data were expressed as mean and standard deviation. For tidal breathing mode, data were described as the median and interquartile range of five representative breaths, which were detected from raw time–TA displacement signal curve, unless otherwise stated. In forced breathing mode, the highest FEV1 + FVC recorded were selected from three measurements according to the spirometry guidelines^2^. The differences between spirometry and MCO parameters were compared using the Mann–Whitney U test. The Spearman’s rho coefficient was used to assess parameter correlations between two methods. *p*-values of < 0.05 were considered statistically significant. All analyses were performed using GraphPad Prism version 5.04 (GraphPad Software, San Diego, CA, the USA).

### Ethics approval

This study was approved by the institutional ethics committee (institutional review board of the University of Tokyo Health Service Center, approved number 17–249), and was conducted in accordance with the Declaration of Helsinki.

### Consent to participate

All participants provided written informed consent for their participation in the study.

## Results

### Demographic characteristics and spirometric data of participants

The demographic characteristics and spirometric data of participants are summarized in Table 1. None had a previous history of smoking, chest surgery, and neuromuscular diseases. Of 48 participants, 11 had a history of childhood asthma. However, none showed symptoms of asthma or other respiratory illnesses on the procedure day. The mean BMI was 21 kg/m^2^, and only three participants met the Japan Society for the Study of Obesity (BMI: ≥ 25 kg/m^2^) criteria. None of the participants met the obstructive (FEV1/FVC of < 0.7) or restrictive (VC of < 80% of the predicted value) ventilatory impairment criteria. There was no significant difference in terms of demographic characteristics and spirometric data between participants with and without a history of childhood asthma (Supplementary Table S1).

**Table 1:** Participants’ characteristics.

	*n* = 48
**Physical examination data**	
Age (year)	19 (3)
Body height (cm)	170 (6.4)
Body weight (kg)	60 (7.2)
Body mass index (kg/m^2^)	21 (2.4)
Arterial oxygen saturation of pulse oximetry (%)	97 (1)
Pulse rate	77 (12)
Chest circumference (at maximum inspiration) (cm)	90 (4.5)
Chest circumference (at maximum expiration) (cm)	84 (4.6)
**Spirometry data**	
VC (L)	4.32 (0.64)
VC, % of predicted	96.0 (12.0)
TV (L)	0.81 (0.36)
FVC (L)	4.34 (0.74)
FVC, % of predicted	96.2 (14.4)
FEV1 (L)	4.00 (0.63)
FEV1 (L), % of predicted	99.0 (13.4)
FEV1/ FVC (%)	92.4 (5.85)
FEV1/ FVC, % of predicted	103 (6.44)
PEF (L/s)	8.35 (1.98)
PEF (L/s), % of predicted	87.5 (20.0)
FEF25–75 (L/s)	5.18 (1.06)
FEF25–75, % of predicted	125 (30.6)
FEF50 (L/s)	5.65 (1.19)
FEF50, % of predicted	90.9 (18.5)
FEF75 (L/s)	3.24 (0.93)
FEF75, % of predicted	89.3 (24.3)
FEF50/FEF75	1.83 (0.40)

Data are expressed as mean (SD).

*VC* vital capacity, *TV* tidal volume, *FVC* forced vital capacity, *FEV1* Forced vital capacity, *PEF* peak expiratory flow, *FEF25–75* forced expiratory flow between 25 and 75% of vital capacity, *FEF50* forced expiratory flow at 50% of forced vital capacity, *FEF75* forced expiratory flow at 25% of forced vital capacity.

### Chest wall motion analysis using the MCO method

**Figure 2:**
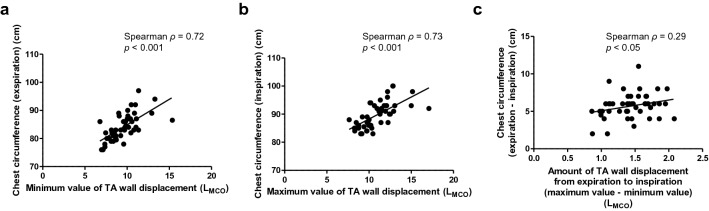
The correlation between chest circumference and MCO parameters. **(a)** Chest circumference at maximum inspiration and maximum value of TA wall displacement, **(b)** chest circumference at maximum expiration and minimum value of TA wall displacement, **(c)** change of chest circumference from maximum inspiration to maximum expiration and the maximum amount of TA wall displacement measured by a slow expiration after the deepest possible inspiration. L_MCO_ on the figure axes indicates the volume which is estimated from TA wall displacement that can be expressed in L by MCO method. *MCO* motion capture system using one-pitch phase analysis, *TA* thoraco-abdominal.

Figure 1b shows an example of surface area (participant No. 1). Separating areas diagonally was hard to do on the current version of equipment due to a technical issue. Thus, to simplify the measuring method, we decided to divide the surface area into eight artificial rectangular compartments. The RC and ABD were divided into four equal compartments (areas A–H). The RCs were divided into the upper (area A + E) and lower (area B + F) half compartments (Fig. 1c). Previous reports showed that chest circumference could predict pulmonary function parameters such as TV and FVC in healthy young participants^30–32^. Hence, we assessed the chest circumference of participants at full inspiration and expiration. As shown in Fig. 2, a significant correlation was observed between chest wall volume measured using the MCO method and the variables of actual chest circumference, which were as follows: chest circumference at the maximum inspiration and maximum value of TA wall displacement (Fig. 2a), chest circumference at the maximum expiration and minimum value of TA wall displacement (Fig. 2b), changes in chest circumference from maximum inspiration to maximum expiration, and maximum amount of TA wall displacement measured during slow expiration after the deepest possible inspiration (Fig. 2c). These results showed that TA wall displacement measured using the MCO method reflects the chest circumference change, which is considered as a surrogate for lung volume parameters including VC, FVC, and total lung capacity^33^. Taken together, TA wall displacement can be considered to partially reflect the lung volume change. Therefore, we considered TA wall displacement as a surrogate marker for volume and TA wall displacement rate for flow in the following procedure.

### Tidal breathing mode analysis

In tidal breathing mode, some of the participants settled down in a stable breathing state after several breaths from the beginning. Therefore, with excluding these unstable ones, five representative breaths of each participant were extracted by the researchers from raw time–TA wall displacement signal curves and were used in the following study, unless otherwise specified. Parameters derived under tidal breathing mode included the following: inspiratory time (Ti), expiratory time (Te), total breath time (Ttot, calculated using the sum of Ti and Te), and respiratory rate (RR, obtained using 60/Ttot), which was calculated with TA wall displacement-over time trace. The amount of TA wall and thoracic wall displacement during restful breathing was defined as TADrb and TWDrb (analogous to conventional tidal volume), respectively. A loop analogous to a conventional tidal flow-volume curve was produced by plotting TA wall displacement rate against TA wall displacement. The peak tidal inspiratory displacement rate (PTIDR), peak tidal expiratory displacement rate (PTEDR), tidal inspiratory displacement rate at 50% of TADrb (TIDR50: analogous to the tidal inspiratory flow when 50% of TV remains in the lung in tidal breathing flow-volume loops), tidal expiratory displacement rate at 50% of TADrb (TEDR50: analogous to the tidal expiratory flow when 50% of TV remains in the lung in tidal breathing flow-volume loops), and its ratio (TIDR50/TEDR50) were identified (Supplementary Figure S2a). The volume of each compartment (i.e., upper RC, lower RC, and ABD) and its percentage contribution to the total chest wall volume were quantified in each tidal breath (Supplementary Figure S2b). The slow VC mode parameter included the maximum amount of TA wall displacement measured using a slow expiration after the deepest possible inspiration (mTADsl: analogous to conventional VC).

**Figure 3:**
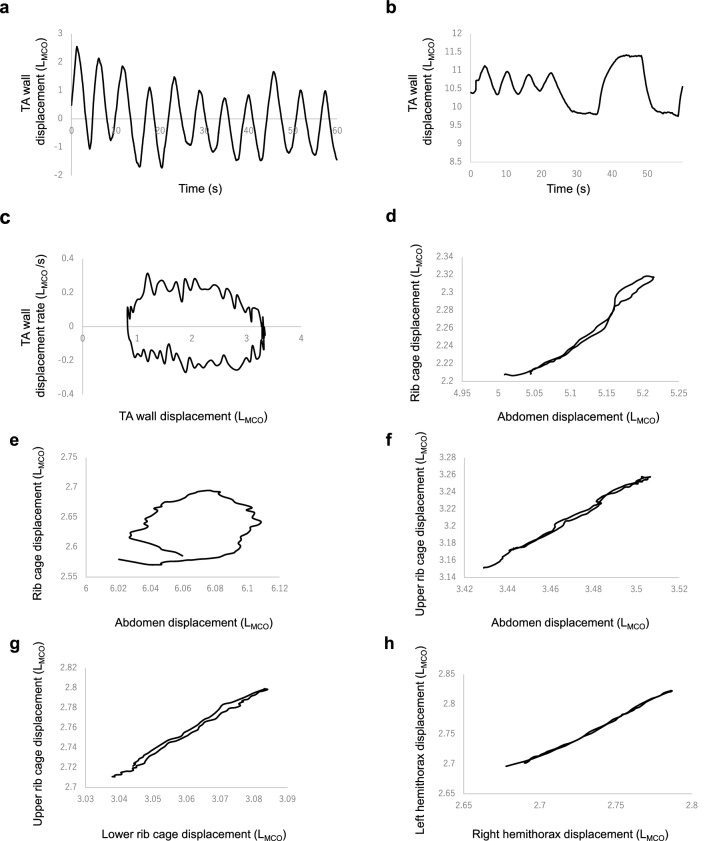
Representative TA wall displacement–time trace by MCO method. Representative TA wall displacement–time trace derived from **(a)** tidal breathing mode, **(b)** slow VC mode (both from participant No. 1), and **(c)** Representative trace of TA wall displacement-TA wall displacement rate derived from tidal breathing mode (analogous to tidal flow-volume curve) (participant No. 8). Representative trace of Konno–Mead diagram derived from tidal breathing mode for **(d)** TAA (total RC vs ABD) (participant No. 8). In this case, TAA is 2.89 degrees; **(e)** TAA (total RC vs ABD) (participant No. 28). In this case, TAA is 47.34 degrees; **(f)** TAA (upper RC vs ABD) (participant No. 32). In this case, TAA is -2.24 degrees; **(g)** RCA (upper RC vs lower RC) (participant No. 15). In this case, RCA is 2.75 degrees; **(h)** HTA (right RC vs left RC) (participant No. 6). In this case, HTA is 0.96 degrees. L_MCO_ on the figure axes indicates the volume which is estimated from TA wall displacement that can be expressed in L by MCO method. *TA* thoraco-abdominal, *VC* vital capacity, *TAA* thoraco-abdominal asynchrony, *RC* rib cage, *ABD* abdomen, *RCA* rib cage asynchrony, *HTA* hemithoracic asynchrony.

All data were successfully calculated under tidal breathing and slow VC modes (Table 2). The median number of breaths calculated using 60/Ttot was 16/min. The representative traces of tidal breathing and slow VC modes are shown in Fig. 3a,b, respectively. A loop analogous to a conventional tidal flow-volume curve was generated (TA wall displacement–TA wall displacement rate loop) (Fig. 3c). In most variables, the median values measured via MCO method were lower than the representative values measured via spirometry, and these results were expected. In the asynchrony study, most participants had closed circles in the Konno–Mead diagrams for each comparison (Figs. 3d–h). The median phase angle value of TAA (upper RC–ABD), TAA (RC–ABD), HTA, and rib cage asynchrony (RCA) were between − 5.05° and 3.86°. Figure S3 shows an example of IPT and EPT. The mean IPT and EPT values of each compartment were nearly zero. Thus, these compartments moved almost synchronous to the counterpart. For the compartmental contribution study (Table 2), the relative contribution of RC was 47.9%, indicating that both RC and ABD contributed subequally to the total chest wall motion. Both sides of the upper and lower RC contributed about 10% to the total chest wall volume, whereas ABD contributed about 25%. These results were substantiated by the overlapping of time–volume curves in both sides of the RC and ABD compartments, although the volume of the right-side ABD compartment was slightly lower than the left side (Supplementary Fig. S4).

**Table 2:** Tidal breathing and slow VC mode parameters measured by MCO.

	*n* = 48
**Chest wall displacement- and time-derived indices**	
TADrb (L_MCO_)	0.18 (0.13–0.25)
TWDrb (L_MCO_)	0.07 (0.05–0.12)
mTADsl (L_MCO_)	1.43 (1.16–1.66)
Ti (s)	1.60 (1.35–2.15)
Te (s)	2.20 (1.85–2.68)
Ttot (s)	3.75 (3.23–5.03)
I/E ratio	1.32 (1.20–1.47)
RR (breath/min)	16.0 (11.9–18.6)
PTIDR	0.24 (0.20–0.28)
PTEDR	0.22 (0.18–0.26)
TIDR50	0.16 (0.14–0.20)
TEDR50	0.14 (0.09–0.18)
**Asynchrony indices**	
**Phase angle**	
TAA (upper RC–ABD) (degree)	−5.05 (−13.9 to 5.81)
TAA (RC–ABD) (degree)	−4.63 (−14.1 to 4.81)
HTA (degree)	2.21 (−0.89 to 3.72)
RCA (upper RC–lower RC) (degree)	3.86 (0.86–7.19)
**Paradox time**	
IPT of upper RC (%)	1.89 (1.54)
IPT of lower RC (%)	2.22 (2.48)
IPT of ABD (%)	0.63 (2.85)
EPT of upper RC (%)	0.76 (0.95)
EPT of lower RC (%)	0.54 (0.86)
EPT of ABD (%)	0.72 (0.80)
**Compartmental contribution indices**	
cRC (area A + B + E + F) vs cABD (area C + D + G + H) (%)	47.9 (47.1–48.8) vs 52.1 (51.2–52.9)
cURC right (area A) vs left (area E) (%)	11.6 (11.3–11.8) vs 11.5 (11.2–11.8)
cLRC right (area B) vs left (area F) (%)	12.4 (12.2–12.5) vs 12.5 (12.3–12.6)
cHT right (area A + B) vs left (area E + F) (%)	23.9 (23.5–24.4) vs 24.0 (23.5–24.4)
cABD right (area C + D) vs left (area G + H) (%)	25.6 (25.3–26.1) vs 26.4 (26.1–26.9)

Data are expressed as median (interquartile range) unless otherwise specified. For paradox time indices, data are expressed as mean (SD). L_MCO_ on the figure axes indicates the volume which is estimated from TA wall displacement that can be expressed in L by MCO method.

*MCO* motion capture using one-pitch phase analysis, *TADrb* thoraco-abdominal wall displacement measured by restful breathing, *TWDrb* thoracic wall displacement measured by restful breathing, *mTADsl* the maximum amount of thoraco-abdominal wall displacement measured by a slow expiration after the deepest possible inspiration, *Ti* inspiratory time, *Te* expiratory time, *Ttot* total breath time, *I/E* inspiration time/expiration time, *RR* respiratory rate, *PTIDR* peak tidal inspiratory displacement rate, *PTEDR* peak tidal expiratory displacement rate, *TIDR50* tidal inspiratory displacement rate at 50% of TADrb, *TEDR50* tidal expiratory displacement rate at 50% of TADrb, *TAA* thoraco-abdominal asynchrony, *RC* rib cage, *ABD* abdomen, *HTA* hemi-thoracic asynchrony, *RCA* rib cage asynchrony, *IPT* inspiratory paradox time, *EPT* expiratory paradox time, *cRC* compartmental contribution of the rib cage to the total thoraco-abdominal wall movement, *cABD* compartmental contribution of the abdomen to the total thoraco-abdominal wall movement, *cURC* compartmental contribution of the upper rib cage to the total thoraco-abdominal wall movement, *cLRC* compartmental contribution of the lower rib cage to the total thoraco-abdominal wall movement, *cHT* compartmental contribution of the hemithorax to the total thoraco-abdominal wall movement, *cABD* compartmental contribution of the abdomen to the total thoraco-abdominal wall movement.

### Forced breathing mode analysis

**Figure 4:**
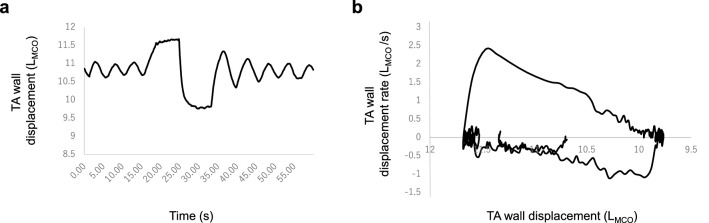
Representative curve derived from forced breathing mode. **(a)** Representative TA wall displacement–time trace derived from forced breathing mode. **(b)** Representative trace of TA wall displacement-TA wall displacement rate derived from forced breathing mode (analogous to conventional flow-volume curve). All data are from participant No. 1. L_MCO_ on the figure axes indicates the volume which is estimated from TA wall displacement that can be expressed in L by MCO method. *TA* thoraco-abdominal.

The forced breathing mode parameters included the maximum amount of TA wall displacement measured during forced expiration after the deepest possible inspiration (mTADf, analogous to conventional FVC), amount of TA wall displacement within 1 s measured during forced expiration after the deepest possible inspiration (fTAD1, analogous to conventional FEV1), fTAD1/mTADf (analogous to conventional FEV1/FVC), peak expiratory flow derived using TA wall displacement rate–TA wall displacement curve (TADpf, analogous to conventional peak expiratory flow), forced expiratory flow rate at 50% of mTADf (TADff50, analogous to FEF50), and forced expiratory flow rate at 25% of mTADf (TADff75, analogous to FEF75). Using the forced breathing maneuver, a loop analogous to typical Tiffeneau curves and flow-volume curves were generated by plotting TA wall displacement rates against TA wall displacement (Fig. 4a,b). With the use of these curves, forced breathing parameters were evaluated (Table 3). In four participants, TADpf, TADff50, and TADff75 were not evaluated due to unstable breathing. The median mTADsl (analogous to slow VC: 1.43 L_MCO_) and mTADf (analogous to FVC: 1.48 L_MCO_) values were almost equivalent, as with the spirometry data (mean slow VC: 4.32 L; mean FVC: 4.34 L).

**Table 3:** Forced breathing parameters measured by MCO.

	*n* = 48
mTADf (L_MCO_)	1.48 (1.26–1.62)
fTAD1 (L_MCO_)	0.84 (0.57–1.10)
fTAD1/mTADf (%)	59.7 (44.0–75.4)
TADpf^a^	1.57 (1.16–2.09)
TADff_50_^a^	1.06 (0.75–1.48)
TADff_75_^a^	0.40 (0.25–0.76)
TADff_50_/TADff_75_^a^	2.19 (1.77–3.21)

Data are expressed as median (interquartile range). L_MCO_ on the figure axes indicates the volume which is estimated from TA wall displacement that can be expressed in L by MCO method.

*MCO* motion capture using one-pitch phase analysis, *mTADf* the maximum amount of thoraco-abdominal wall displacement measured by a forced expiration after the deepest possible inspiration, *fTAD1* the amount of thoraco-abdominal wall displacement in first one second measured by a forced expiration after the deepest possible inspiration, *TADpf* peak expiratory flow derived from flow–thoraco-abdominal wall displacement curve, *TADff50* forced expiratory flow rate at 50% of mTADf, *TADff75* forced expiratory flow rate at 25% of mTADf.

^a^TADpf, TADff50, TADff75, and TADff50/TADff75 were not evaluated due to unstable breathing in four participants.

**Figure 5:**
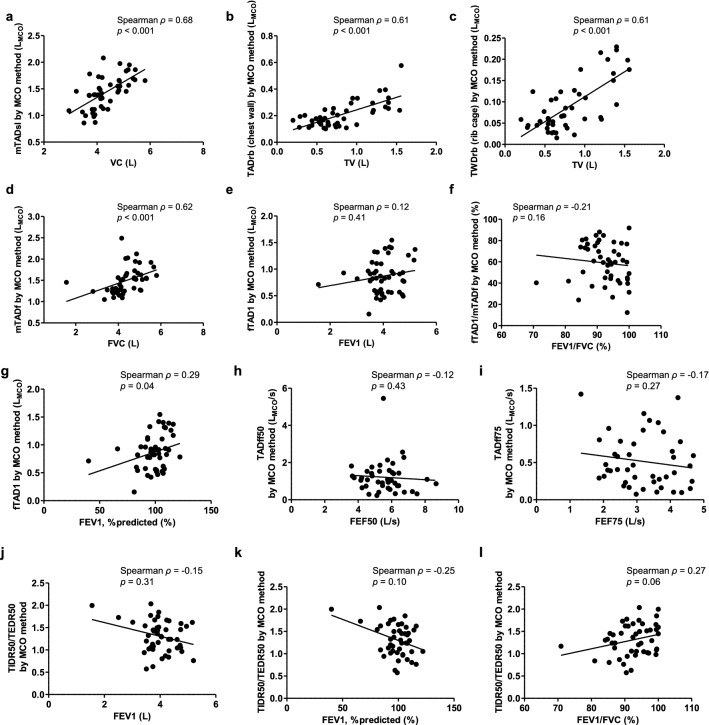
Correlation between spirometric and MCO parameters. **(a)** VC and mTADsl, **(b)** TV and TADrb (total chest wall), **(c)** TV and TWDrb (rib cage), **(d)** FVC and mTADf, **(e)** FEV1 and fTAD1, **(f)** FEV1/FVC and fTAD1/mTADf, **(g)** percent predicted value of FEV1 and fTAD1, **(h)** FEF50 and TADff50, **(i)** FEF75 and TADff75, **(j)** FEV1 and TIDR50/TEDR50, **(k)** percent predicted value of FEV1 and TIDR50/TEDR50, **(l)** FEV1/FVC and TIDR50/TEDR50. L_MCO_ on the figure axes indicates the volume which is estimated from TA wall displacement that can be expressed in L by MCO method. *MCO* motion capture system using one-pitch phase analysis, *VC* vital capacity, *mTADsl* maximum amount of thoraco-abdominal wall displacement measured by a slow expiration after the deepest possible inspiration, *TV* tidal volume, *TADrb* amount of TA wall displacement during restful breathing, *TWDrb* amount of thoracic wall displacement during restful breathing, *FVC* forced vital capacity, *mTADf* maximum amount of thoraco-abdominal wall displacement measured by a forced expiration after the deepest possible inspiration, *FEV1* expiratory forced volume in the first second, *fTAD1* amount of thoraco-abdominal wall displacement in first one second measured by a forced expiration after the deepest possible inspiration, *FEF50* and *FEF75* forced expiratory flow at 50% and 25% of forced vital capacity, *TADff50* and *TADff75* forced expiratory flow rate at 50% and 25% of mTADf, *TIDR50* tidal inspiratory displacement rate at 50% of thoraco-abdominal wall displacement, *TEDR50* tidal expiratory displacement rate at 50% of thoraco-abdominal wall displacement.

Based on the data obtained via spirometry and MCO method, a significant positive correlation was observed between VC and mTADsl (Spearman’s *ρ* = 0.68, *p* < 0.0001) (Fig. 5a), TV and TADrb (Spearman’s *ρ* = 0.61, *p* < 0.0001) (Fig. 5b), TV and TWDrb (Spearman’s *ρ* = 0.61, *p* < 0.0001) (Fig. 5c), and mTADf and FVC (Spearman’s *ρ* = 0.62, *p* < 0.0001) (Fig. 5d). However, the spirometric parameters of obstructive ventilatory impairment were not correlated with MCO parameters except for fTAD1 and the percentage of predicted FEV1 value, which revealed a weak positive correlation (Fig. 5e–l).

For the aforementioned parameters, no statistically significant differences were observed between participants with and without a previous history of asthma (Supplementary Tables S2 and S3).

## Discussion

To the best of our knowledge, this study first analyzed chest wall motion using the MCO method. Based on this feasibility research, diverse parameters could be calculated under tidal breathing, slow VC, and forced breathing modes in almost all participants. The mTADsl, mTADf, TADrb, and TWDrb values were positively correlated with the corresponding spirometric parameters. Furthermore, chest wall asynchrony and compartmental contribution indices were successfully measured using the MCO method. Further, each compartment of the chest wall moved synchronously in healthy participants. Thus, MCO can be a promising diagnostic tool for evaluating chest wall motion and pulmonary function.

The MCO method has the following advantages: First, this method can display three-dimensional, regional TA wall movement data output in real-time. Thus, it gives a deep insight into the breathing mechanics which cannot be measured by spirometry. Second, it is a non-contacting technique to estimate lung volume changes. Compared to spirometry, it has the advantage of infection control and gives less stress to a patient since it does not require a mouthpiece and a nose-clip. Third, compared to radiological imaging techniques including computed tomography, it uses visible light imaging technology so that avoiding radiation exposure. Fourth, it does not require any disposables, therefore, running costs can be reduced. Fifth, its compact and simple structure has the potential to measure chest wall volume at patients’ bedside and in a supine position by projecting the grating pattern from above, reducing the need of moving a patient to the measurement site and position. Sixth, compared to most other motion capture systems including OEP, its marker-less nature saves time and trained personnel during an examination, leading to a higher possibility of application in daily clinical practice. On the contrary, potential disadvantages of the MCO method include the following: First, at present, it cannot fully cover entire chest wall area, therefore, the measured value by the MCO method is smaller than that of spirometry. Second, currently, we do not have a dedicated program for the chest wall motion analysis and need to calculate the respiratory parameters manually. Third, the definition of RC and ABD are not as rigorously based on anatomical structures as marker-attaching methods including OEP. Fourth, the brightness of the room and the lustre of the wall behind affect the results. Finally, it may be inferior in detecting the lateral area motion of the body.

The chest wall motion analyses using Moiré topography had been already conducted in 1984^34,35^. Peacock et al. analyzed the TA wall volume change during breathing using a vertical pattern projected on the trunk and showed close values to the volume measured by water displacement^34^. Morgan et al. applied the same method to measure chest wall movement and demonstrated a good correlation with spirometry^35^. However, in the former study, the volume was measured by two cameras and two projectors in upright positioning. It was measured with the use of one camera and two projectors in supine positioning in the latter study. These methods are slightly more complicated than the MCO method (composed of one camera and projector), and measured volume in different positioning from that of spirometry. Moreover, these methods took about 30 min to analyze each image. The MCO method has the advantage that it can display a result in real-time.

Spirometry is a widely used screening tool for measuring pulmonary function. However, it is challenging to perform on some patients such as young children, those with dyspnea and dementia as they cannot cooperate. In addition, spirometry requires a mouthpiece and nose clip, which cause unnatural breathing and may alter breathing pattern, thereby affecting examination results^36,37^. The MCO method is a non-contacting, easy to use method, and it requires less participant cooperation during the analysis of tidal breathing, thereby facilitating a more natural breathing. Thus, it may be more advantageous than spirometry among participants who cannot fully cooperate. In addition, the non-contacting nature of MCO method is beneficial in terms of infection control since mouthpieces and spirometry tubing may be contaminated with microorganisms^38,39^, particularly during the coronavirus disease 2019 pandemic. Moreover, its compact structure can easily facilitate data acquisition at patients’ bedside. In the future study, we plan to project a grating pattern from above the participants, so that we can measure the chest wall motion of a person who cannot sit such as an infant, patients who are unable to communicate, and critically ill patients. In relation to these reasons, MCO method can simply enable chest wall motion analysis; therefore, it can be acceptable in clinical practice.

A number of chest wall displacement- and time-derived parameters were calculated under tidal breathing and slow VC modes. The median Ttot value was 3.75 s, indicating that the median RR was 16/min. This result was in accordance with that of a previous study, which showed the normal RR range in healthy adolescents (median RR in children aged 18 years: around 16 cycles per minute)^40^. Moreover, the Ti (1.60 s) and Te (2.20 s) ratios are also agrees with data obtained from healthy controls in a previous study^26,41^. TWDrb (analogous to tidal volume), TADrb (analogous to total chest wall volume), and mTADsl (analogous to slow VC) were positively correlated with conventional TV and slow VC. These results showed that MCO is a useful method for assessing lung volume in healthy participants. However, in the current study, the absolute values of these parameters were lower than those of spirometry. Moreover, our study showed moderate correlation for VC, FVC, and TV. In contrast, the volumetric variables of OEP showed nearly equal absolute spirometry parameter values, with a mean VC difference of 68 mL, between the two methods and showed a strong correlation ^42–44^. This finding is mainly attributed to the setting of surface area for data collection. In our study, the region from the clavicle to the umbilicus in a vertical direction and from the right to the left nipple in a horizontal direction was defined as surface area (Fig. 1c), which was smaller than that of OEP. In addition, participants were instructed to sit upright and keep their back on the wall. Thus, we may fail to detect outer area movement of the body surface and it might not be effective in detecting posterior wall movements of the chest. The use of multiple projectors and cameras from other directions may improve the detection of lateral and posterior movements. However, the whole system will become more complex. On the contrary, parameters of obstructive disorders did not show a significant correlation except for the % predicted value of FEV1 and fTAD1. These parameters reflect the inner diameter of the respiratory tract. Therefore, it may be difficult to estimate them by measuring outer surface body movement alone. Further investigation will be needed to detect surrogate parameters for obstructive disorders by the MCO method.

Regional chest wall asynchrony assessment is important in evaluating breathing dynamics. In healthy participants, the RC and ABD moved synchronously with only little distortions during spontaneous breathing^21,41,45^. By contrast, TAA, an uncoordinated movement of RC and ABD during respiration, occurs in respiratory and neuromuscular disorders^5^. It is correlated with airflow obstruction, leading to breathlessness and exercise intolerance^29,46,47^. Since paradoxical movement of the chest wall was associated with the early indicators of respiratory impairment in previous studies, we investigated asynchrony indices using the MCO method with chest wall kinematics proposed by Konno and Mead^22^. Thus, the median phase angle values of TAA were − 5.05° for upper RC–ABD and − 4.63° for total RC–ABD. A negative angle (counter − clockwise direction of loop) indicates that the ABD compartment (diaphragm) leads to the RC, as commonly observed in normal quiet breathing^48^, and these results are in line with the TAA value of control participants in a previous study that analyzed by OEP, in which the phase angle ranged from − 6.6° to 14.0°^5,26,29,41^. The mean IPT and EPT values of each compartment ranged from 0.54% to 2.22% in this study. These findings are in accordance with those observed in control participants (about 5%–10%, < 20%)^25,26,28,29,41^, indicating that the RC and ABD had an almost concordant movement to the total chest wall motion. Taken together, MCO can validate synchronous respiratory motion of the TA wall among healthy individuals during quiet breathing.

Regarding compartmental volumes, the RC (47.9%) and ABD (52.1%) contributed almost equally to the total chest wall movement. However, ABD had a slightly higher contribution. These results are congruent with the those of Porras D^23^ and Fregonezi^26^. However, the findings were inconsistent with those of Priori^25^ and Layton^49^, which showed that RC had a higher contribution than ABD. The discrepancy in results may be attributed to difference in age, sex, physique, and definition of each compartment area. For laterality comparison, both sides of each compartment contributed almost equally to the total movement. In addition, all Konno–Mead diagrams of HTA (median phase angle: 2.21°) and RCA (median phase angle: 3.86°) had almost a straight-line shape. Therefore, MCO method can successfully detect bilateral synchronous movement of the chest wall in healthy participants. Meanwhile, in the comparison of the right and left sides of the compartments, the volume of the right-side ABD compartment was slightly lower than the left side as shown in Supplementary Figure S4. This result was similar for other participants. We speculate that this was caused by the camera lens distortion. In the usual measurement, we focus on having a wide lens for the camera and short-range in order to put the middle part of the lens in the measuring area so that the distortion does not happen. However, we used the system which was not specialized hardware created for chest wall motion analysis of humans, therefore, it cannot be denied that the measurement area might have been located at the edge of the camera view. If this would happen, the volume of either the left or right side of some parts of the measurement area might be decreased due to the lens distortion. Further work is needed to confirm the speculation.

Under forced breathing mode, mTADf was positively correlated with FVC, which is an analogous parameter in spirometry. However, other parameters were not correlated with the counterparts of spirometry, except for fTAD1 and the percentage of predicted FEV1 value. This is partly explained by the participants’ motion artifact causing noise to MCO data since maximal breathing out effort is required in this mode. The use of body-fixing tools including seatbelt might improve data stability.

The Kinect is a publicly available, inexpensive and portable time-of-flight depth sensor that can be used for a motion capture system. Based on these advantages, some studies evaluate the chest wall motion using Kinect. Harte et al. evaluated lung volume measured by spirometry and Kinect-based method simultaneously and showed a good correlation between the two methods^10^. However, they used four optical systems and captured chest wall motion from both anteroposterior and crosswise directions. Moreover, the participants performed three times measurement of Kinect-based chest wall motion analysis, therefore, they might get used to the procedure. These factors may lead to efficient capturing of chest wall movement and affect the result of the high correlational relationship between the two methods. Povšič et al. also measured TA wall motion by Kinect-based method^13^. They employed five markers (two in clavicle, one in sternum, and two in ilium) on the body surface to exclude breathing unrelated movement. Although attaching markers will be beneficial in increasing accuracy for volume measurement, this may be time-consuming and complicate the system. We conceive that our research has the advantage of simplicity because of its non-contacting nature. Yu et al.^11^ and Sharp et al.^12^ used only one Kinect depth camera with a non-contacting manner in capturing chest wall motion in their study, and they showed a high correlation of respiratory volume measured by between spirometry and Kinect-based method. There are several reasons for the discrepancy between these studies and our study in terms of the correlation of two analyzing methods. First, in the former study, twelve participants performed sixteen rounds of respiratory measurement. Therefore, as described above, the participants’ familiarity with the measurement method may affect the results. Second, the participants carried out spirometry and Kinect-based system simultaneously in both studies. Thus, the volume values were expected to be close. On the contrary, in our study, the participants performed spirometry and MCO method separately. When performing spirometry, the participants did not prohibit moving their body, while they were asked to sit upright with their back on the chair when they perform the MCO method. Third, the analyzing area of chest wall motion might differ. The measuring body area seemed to be larger enough so that it covered almost all areas of the anterior chest wall in the study of Yu et al. and Sharp et al., while the surface area for measuring in our study smaller as shown in Fig. 1b. These factors might result in a lower correlation between spirometry and MCO method.

Previous studies have shown significant changes in wall displacement- and time-derived parameters (i.e., peak tidal inspiratory/expiratory flow)^50^ and asynchrony parameters (i.e., TAA, IPT, and EPT)^25,26,29^ between patients with pulmonary diseases and controls. However, in the current study, no statistically significant differences have been observed in MCO parameters between participants with and without a previous history of asthma. The discrepancies may be caused by the fact that in spirometry, the preserved pulmonary function of participants with and without a previous history of asthma did not differ. Hence, further studies targeting patients with chronic lung diseases who presented with decreased pulmonary function should be conducted.

The current study had several limitations. First, only a small number of students participated. Second, the efficacy of the MCO technique was only validated among young, healthy, male participants, who were bare-chested in a seated position during examination. Therefore, our findings could not be generalized to other populations such as women, elderly individuals, clothes-wearing participants, standing or lysing positioning, and patients with pulmonary and other diseases. In addition, intra-subject reliability should be evaluated. Third, there were only few obese participants, with a mean BMI of 21 kg/m^2^, and only three participants met the criteria on obesity. In obese participants, anatomical landmarks used for the analysis of surface area are challenging to identify. Moreover, Bracelet et al. have reported that obesity is associated with significant decrease in RC volume and increase in ABD volume measured via OEP^51^. Thus, the effect of physique on MCO parameters should be validated. Fourth, all participants in this study performed the MCO method for the first time, therefore, unstable breathing manoeuvres and body movement due to unfamiliarity with this procedure could bring artefacts. Fifth, we measured the movement towards the sensor plane and did not take other directions of movement into account. On the contrary, Solav et al. applied 52 reflective markers attached to the body surface and showed detailed chest wall kinematics^52^. They evaluated four time-variant scalar parameters including outward translation, rotation angle, area change, and shape distortion. As a result, patients with neuromuscular diseases exhibited asynchronous and paradoxical movements of some local areas of the thorax and abdomen, which the traditional volumetric model of OEP could not detect. However, we place emphasis on the simplicity of the measuring method since we plan to apply the MCO method in daily clinical settings. In order to do this, it is necessary to examine many participants in a short time. Thus, the marker-less nature of the MCO method is crucial, even at the expense of some degree of accuracy. Finally, the anatomical definition of upper RC, lower RC, and ABD for MCO method is not well-established. In OEP, the chest wall area covers entire compartment and is divided into three compartments (pulmonary RC, abdominal RC, and ABD) based on the 89 retroreflective markers placed on specific anatomical points in the thorax and ABD^27,44, 49,53^. These methods are validated both in healthy participants and those with diseases, and they have good reproducibility. In the current study, the surface area was defined as an artificial rectangular area and did not include whole chest wall. Besides, it was divided into two compartments (RC and ABD), and the definition of area differed from that of OEP. The anatomical definition of the chest wall compartment assessed using the MCO method should be optimized by including a large number of participants.

## Conclusions

This proof-of-concept study provided novel and important data on the use of MCO method in chest wall motion analysis among healthy participants. Compared with conventional motion capture systems, this method could be used as an alternative diagnostic tool to spirometry and could provide an insight into chest wall motion kinematics in a simpler way. However, further studies with a larger cohort of healthy participants and individuals with pulmonary or neuromuscular diseases must be conducted.

## Supplementary Information

Supplementary Figure S1.

Supplementary Figure S2.

Supplementary Figure S3.

Supplementary Figure S4.

Supplementary Table S1.

Supplementary Table S2.

Supplementary Table S3.

## Data Availability

Individual data used for this study are not publicly available due to confidentiality agreement but are available from the corresponding author on reasonable request as long as the request meets the ethics.

## Abbreviations

ABD: Abdomen
BMI: Body mass index
COPD: Chronic obstructive pulmonary disease
EPT: Expiratory paradox time
FEF25-75: Forced expiratory flow between 25 and 75% of forced vital capacity
FEF50: Forced expiratory flow at 50% of forced vital capacity
FEF75: Forced expiratory flow at 25% of forced vital capacity
FEV1: Expiratory forced volume in the first second
fTAD1: Amount of thoraco-abdominal wall displacement in first one second measured by a forced expiration after the deepest possible inspiration
FVC: Forced vital capacity
HTA: Hemithoracic asynchrony
IPT: Inspiratory paradox time
MCO: Motion capture system using one-pitch phase analysis
mTADf: Maximum amount of thoraco-abdominal wall displacement measured by a forced expiration after the deepest possible inspiration
mTADsl: Maximum amount of thoraco-abdominal wall displacement measured by a slow expiration after the deepest possible inspiration
OEP: Optoelectronic plethysmography
PEF: Peak expiratory flow
PTEDR: Peak tidal expiratory displacement rate
PTIDR: Peak tidal inspiratory displacement rate
RC: Rib cage
RCA: Rib cage asynchrony
RR: Respiratory rate
TA: Thoraco-abdominal
TAA: Thoraco-abdominal asynchrony
TADff50: Forced expiratory flow rate at 50% of the maximum amount of thoraco-abdominal wall displacement measured by a forced expiration after the deepest possible inspiration
TADff75: Forced expiratory flow rate at 25% of the maximum amount of thoraco-abdominal wall displacement measured by a forced expiration after the deepest possible inspiration
TADpf: Peak expiratory flow derived from thoraco-abdominal wall displacement rate–thoraco-abdominal wall displacement curve
TADrb: Amount of thoraco-abdominal wall displacement during restful breathing
Te: Expiratory time
TEDR50: Tidal expiratory displacement rate at 50% of thoraco-abdominal wall displacement
Ti: Inspiratory time
TIDR50: Tidal inspiratory displacement rate at 50% of thoraco-abdominal wall displacement
Ttot: Total breath time
TV: Tidal volume
TWDrb: Amount of thoracic wall displacement during restful breathing
VC: Vital capacity
