# Supplementary material for: Measurement of chest wall motion using a motion capture system with the one-pitch phase analysis method
Source: Sci Rep. 2021 Nov 2;11:21497. doi: 10.1038/s41598-021-01033-8 (PMC8563798; doi:10.1038/s41598-021-01033-8)
Supplement: Supplementary file 1 — Supplementary Figure S1. [file 41598_2021_1033_MOESM1_ESM.pdf]

Title:

Measurement of Chest Wall Motion Using a Motion Capture System with the One-pitch Phase Analysis Method

Authors' full names:

Hiroyuki Tamiya, M.D., Ph.D. [1)], Akihisa Mitani*, M.D., Ph.D. [1, 2)], Hideaki Isago, M.D., Ph.D. [1,3)], Taro Ishimori, M.D., Ph.D. [1)], Minako Saito, M.D., Ph.D. [1, 2)], Taisuke Jo, M.D., Ph.D. [1,2)], Goh Tanaka, M.D., Ph.D. [1)], Shintaro Yanagimoto, M.D., Ph.D. [4)], Takahide Nagase, M.D., Ph.D. [1)]

***Corresponding author**

Authors' affiliations:

[1)] The Department of Respiratory Medicine, The University of Tokyo Hospital, 7-3-1, Hongo, Bunkyo-ku, Tokyo 113-8655, Japan

[2)] Health Service Center, The University of Tokyo, 7-3-1 Hongo, Bunkyo-ku, Tokyo, 113-8655, Japan

[3)] The Department of Clinical Laboratory, The University of Tokyo Hospital, 7-3-1, Hongo, Bunkyo-ku, Tokyo 113-8655, Japan

[4)] The Division for Health Service Promotion, The University of Tokyo, 7-3-1, Hongo, Bunkyo-ku, Tokyo 113-8655, Japan

Corresponding author full contact details:

Akihisa Mitani, M.D., Ph.D

Address: The Department of Respiratory Medicine, The University of Tokyo Hospital, 7-3-1, Hongo, Bunkyo-ku, Tokyo, 113-8655, Japan

Email: mitania-int@h.u-tokyo.ac.jp

TEL: +81-3-3815-5411

Fax: +81-3-3814-0021

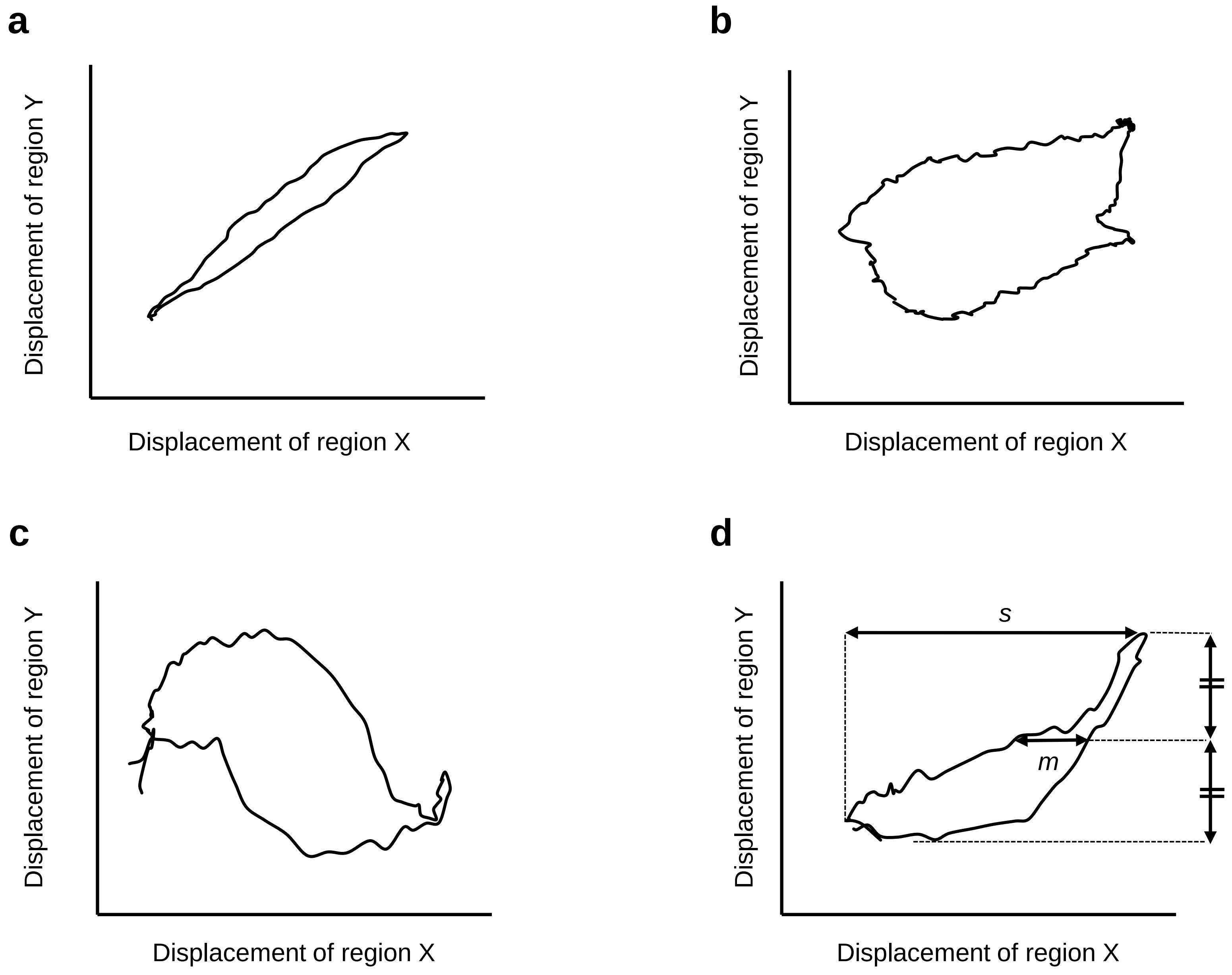

Supplementary Figure S1.

Supplementary Figure S1. Examples and interpretation of Konno–Mead diagram.

Examples and interpretation of Konno–Mead diagram. **(a)** almost synchronous movement (elliptical circle upward to the right: participant No. 41) **(b)** asynchronous movement (nearly perfect circle: participant No. 19) **(c)** paradoxical movement (circle downward to the right: participant No. 33). (d) The level of asynchrony (phase angle) is calculated as arcsin (*m*/*s*) based on Konno–Mead diagram (participant No. 14). *m* = the width of the loop at 50% of y-axis displacement; *s* = the range of x-axis displacement.
