# Supplementary material for: Measurement of chest wall motion using a motion capture system with the one-pitch phase analysis method
Source: Sci Rep. 2021 Nov 2;11:21497. doi: 10.1038/s41598-021-01033-8 (PMC8563798; doi:10.1038/s41598-021-01033-8)
Supplement: Supplementary file 2 — Supplementary Figure S2. [file 41598_2021_1033_MOESM2_ESM.pdf]

Title:

Measurement of Chest Wall Motion Using a Motion Capture System with the One-pitch Phase Analysis Method

Authors' full names:

Hiroyuki Tamiya, M.D., Ph.D. [1)], Akihisa Mitani*, M.D., Ph.D. [1, 2)], Hideaki Isago, M.D., Ph.D. [1,3)], Taro Ishimori, M.D., Ph.D. [1)], Minako Saito, M.D., Ph.D. [1, 2)], Taisuke Jo, M.D., Ph.D. [1,2)], Goh Tanaka, M.D., Ph.D. [1)], Shintaro Yanagimoto, M.D., Ph.D. [4)], Takahide Nagase, M.D., Ph.D. [1)]

***Corresponding author**

Authors' affiliations:

[1)] The Department of Respiratory Medicine, The University of Tokyo Hospital, 7-3-1, Hongo, Bunkyo-ku, Tokyo 113-8655, Japan

[2)] Health Service Center, The University of Tokyo, 7-3-1 Hongo, Bunkyo-ku, Tokyo, 113-8655, Japan

[3)] The Department of Clinical Laboratory, The University of Tokyo Hospital, 7-3-1, Hongo, Bunkyo-ku, Tokyo 113-8655, Japan

[4)] The Division for Health Service Promotion, The University of Tokyo, 7-3-1, Hongo, Bunkyo-ku, Tokyo 113-8655, Japan

Corresponding author full contact details:

Akihisa Mitani, M.D., Ph.D

Address: The Department of Respiratory Medicine, The University of Tokyo Hospital, 7-3-1,

Hongo, Bunkyo-ku, Tokyo, 113-8655, Japan

Email: mitania-int@h.u-tokyo.ac.jp

TEL: +81-3-3815-5411

Fax: +81-3-3814-0021

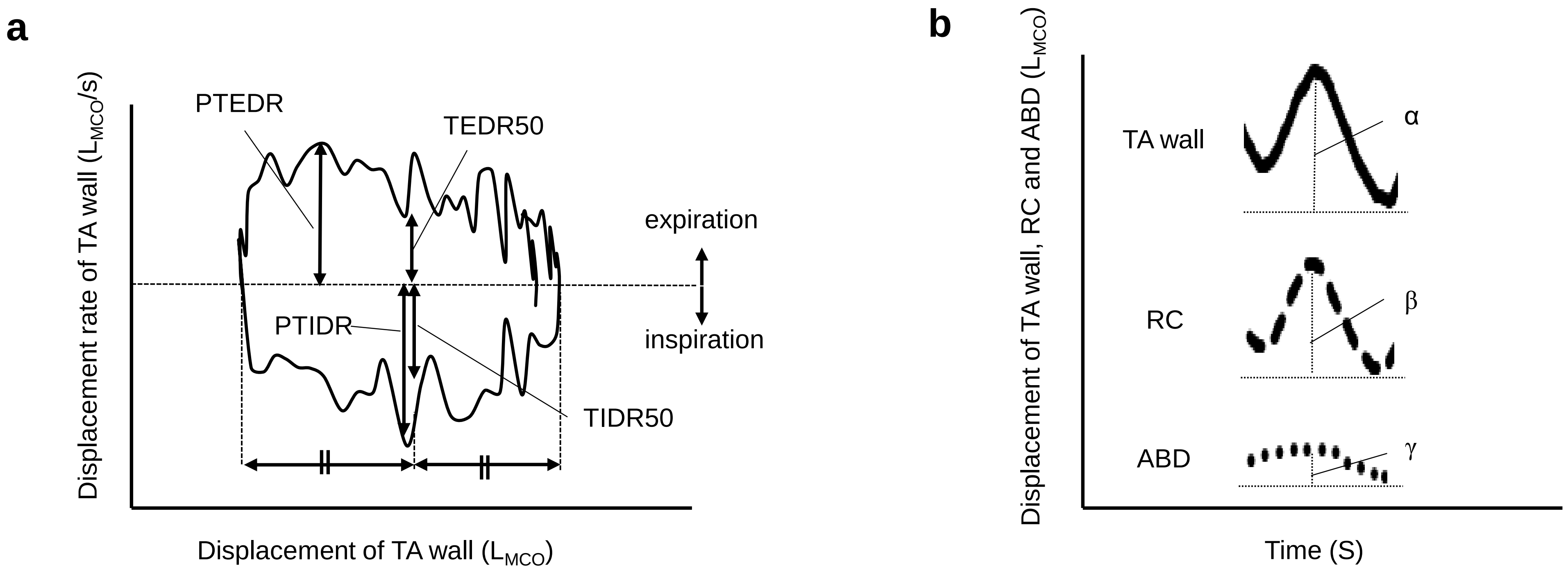

Supplementary Figure S2.

Supplementary Figure S2. Example of loop for a conventional tidal flow-volume curve and compartmental contribution analysis generated by MCO method.

Example of loop for a conventional tidal flow-volume curve and compartmental contribution analysis generated by MCO method. **(a)** Example of a loop analogous to a conventional tidal flow-volume curve which is produced by plotting TA wall displacement rate against TA wall displacement, in which TIDR50 and TEDR50 are calculated. **(b)** Example of time-displacement plot of TA wall (solid line), ABD (dashed line), and RC (dotted line) derived from a single breath (participant No.1). Dividing peak RC displacement (γ) by peak TA wall displacement (α) represents compartmental contribution of the RC. Similarly, dividing peak ABD displacement (β) by peak TA wall displacement (α) represents the compartmental contribution of the ABD. L_{MCO} on the figure axes indicates the volume which is estimated from TA wall displacement that can be expressed in L by MCO method.

Abbreviations: *MCO* motion capture system using one pitch phase analysis, *TA* thoraco-abdominal, *ABD* abdomen, *RC* rib cage, *PTIDR* peak tidal inspiratory displacement rate, *PTEDR* peak tidal expiratory displacement rate, *TIDR50* tidal inspiratory displacement rate at 50% of thoraco-abdominal wall displacement, *TEDR50* tidal expiratory displacement rate at 50% of thoraco-abdominal wall displacement
