# Supplementary material for: Measurement of chest wall motion using a motion capture system with the one-pitch phase analysis method
Source: Sci Rep. 2021 Nov 2;11:21497. doi: 10.1038/s41598-021-01033-8 (PMC8563798; doi:10.1038/s41598-021-01033-8)
Supplement: Supplementary file 3 — Supplementary Figure S3. [file 41598_2021_1033_MOESM3_ESM.pdf]

Title:

Measurement of Chest Wall Motion Using a Motion Capture System with the One-pitch Phase Analysis Method

Authors' full names:

Hiroyuki Tamiya, M.D., Ph.D. [1)], Akihisa Mitani*, M.D., Ph.D. [1, 2)], Hideaki Isago, M.D., Ph.D. [1,3)], Taro Ishimori, M.D., Ph.D. [1)], Minako Saito, M.D., Ph.D. [1, 2)], Taisuke Jo, M.D., Ph.D. [1,2)], Goh Tanaka, M.D., Ph.D. [1)], Shintaro Yanagimoto, M.D., Ph.D. [4)], Takahide Nagase, M.D., Ph.D. [1)]

***Corresponding author**

Authors' affiliations:

[1)] The Department of Respiratory Medicine, The University of Tokyo Hospital, 7-3-1, Hongo, Bunkyo-ku, Tokyo 113-8655, Japan

[2)] Health Service Center, The University of Tokyo, 7-3-1 Hongo, Bunkyo-ku, Tokyo, 113-8655, Japan

[3)] The Department of Clinical Laboratory, The University of Tokyo Hospital, 7-3-1, Hongo, Bunkyo-ku, Tokyo 113-8655, Japan

[4)] The Division for Health Service Promotion, The University of Tokyo, 7-3-1, Hongo, Bunkyo-ku, Tokyo 113-8655, Japan

Corresponding author full contact details:

Akihisa Mitani, M.D., Ph.D

Address: The Department of Respiratory Medicine, The University of Tokyo Hospital, 7-3-1, Hongo, Bunkyo-ku, Tokyo, 113-8655, Japan

Email: mitania-int@h.u-tokyo.ac.jp

TEL: +81-3-3815-5411

Fax: +81-3-3814-0021

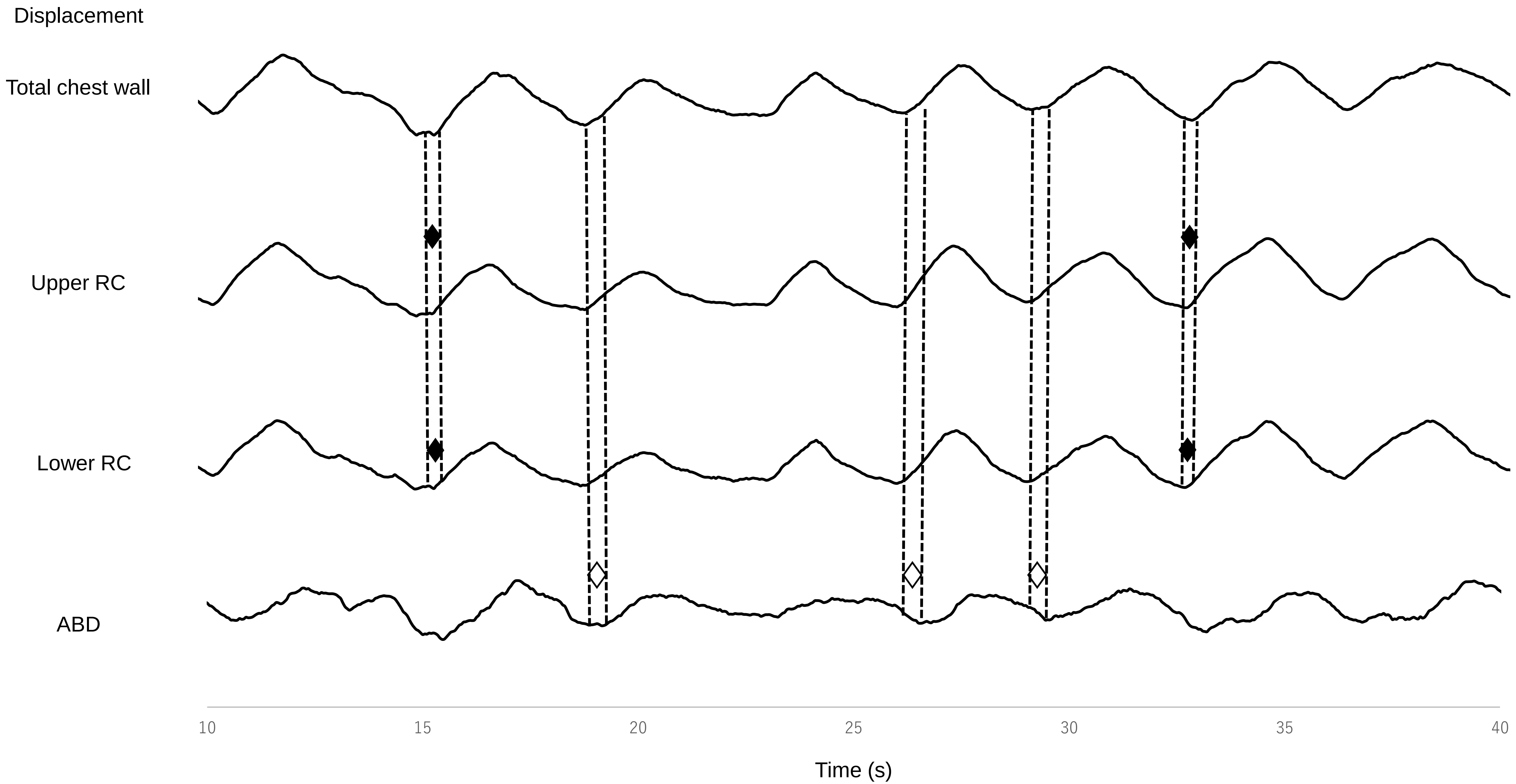

Displacement
Total chest wall
Upper RC
Lower RC
ABD
10
15
20
25
30
35
40
Time (s)

Figure S3.

Supplementary Figure S3. Example of IPT and EPT.

Example of IPT and EPT. Time over the displacement of each compartment of the chest wall are shown (participant No. 28). IPT (◊) and EPT (♦) are defined as the fraction of time during which the compartmental signals moved in opposite direction to the total chest wall displacement signal at the beginning of inspiration and the end of expiration, respectively.

Abbreviations: *IPT* inspiratory paradox time, *EPT* expiratory paradox time
