# Supplementary material for: Measurement of chest wall motion using a motion capture system with the one-pitch phase analysis method
Source: Sci Rep. 2021 Nov 2;11:21497. doi: 10.1038/s41598-021-01033-8 (PMC8563798; doi:10.1038/s41598-021-01033-8)
Supplement: Supplementary file 4 — Supplementary Figure S4. [file 41598_2021_1033_MOESM4_ESM.pdf]

Title:

Measurement of Chest Wall Motion Using a Motion Capture System with the One-pitch Phase Analysis Method

Authors' full names:

Hiroyuki Tamiya, M.D., Ph.D. [1)], Akihisa Mitani*, M.D., Ph.D. [1, 2)], Hideaki Isago, M.D., Ph.D. [1,3)], Taro Ishimori, M.D., Ph.D. [1)], Minako Saito, M.D., Ph.D. [1, 2)], Taisuke Jo, M.D., Ph.D. [1,2)], Goh Tanaka, M.D., Ph.D. [1)], Shintaro Yanagimoto, M.D., Ph.D. [4)], Takahide Nagase, M.D., Ph.D. [1)]

***Corresponding author**

Authors' affiliations:

[1)] The Department of Respiratory Medicine, The University of Tokyo Hospital, 7-3-1, Hongo, Bunkyo-ku, Tokyo 113-8655, Japan

[2)] Health Service Center, The University of Tokyo, 7-3-1 Hongo, Bunkyo-ku, Tokyo, 113-8655, Japan

[3)] The Department of Clinical Laboratory, The University of Tokyo Hospital, 7-3-1, Hongo, Bunkyo-ku, Tokyo 113-8655, Japan

[4)] The Division for Health Service Promotion, The University of Tokyo, 7-3-1, Hongo, Bunkyo-ku, Tokyo 113-8655, Japan

Corresponding author full contact details:

Akihisa Mitani, M.D., Ph.D

Address: The Department of Respiratory Medicine, The University of Tokyo Hospital, 7-3-1,

Hongo, Bunkyo-ku, Tokyo, 113-8655, Japan

Email: mitania-int@h.u-tokyo.ac.jp

TEL: +81-3-3815-5411

Fax: +81-3-3814-0021

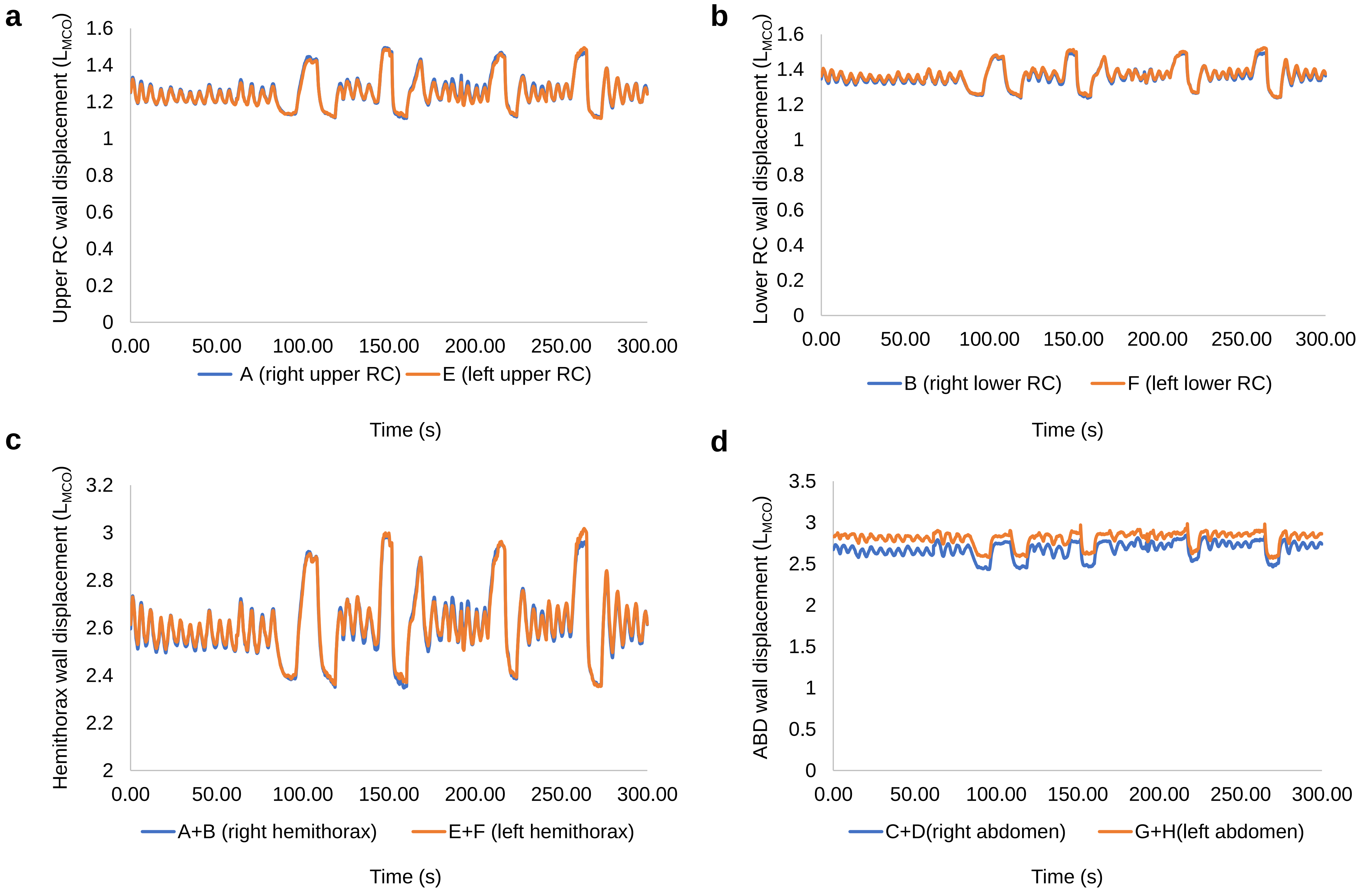

Supplementary Figure S4.

Supplementary Figure S4. Representative case of time–volume curve of the RC and ABD derived from MCO method in tidal breathing maneuver (participant No. 1).

Representative case of time–volume curve of the RC and ABD derived from MCO method in tidal breathing maneuver (participant No. 1). **(a)** Blue (compartment A) and orange (compartment E) lines show the motion of right and left upper RC, respectively. **(b)** Blue (compartment B) and orange (compartment F) lines show the motion of right and left lower RC, respectively. **(c)** Blue (compartment A and B) and orange (compartment E and F) lines show the motion of right and left total hemithorax, respectively. **(d)** Blue (compartment C and D) and orange (compartment G and H) lines show the motion of right and left ABD, respectively. L_{MCO} on the figure axes indicates the volume which is estimated from TA wall displacement that can be expressed in L by MCO method.

Abbreviations: *RC* rib cage, *ABD* abdomen, *MCO* motion capture system using one pitch phase analysis
