# Supplementary material for: Measurement of chest wall motion using a motion capture system with the one-pitch phase analysis method
Source: Sci Rep. 2021 Nov 2;11:21497. doi: 10.1038/s41598-021-01033-8 (PMC8563798; doi:10.1038/s41598-021-01033-8)
Supplement: Supplementary file 5 — Supplementary Table S1. [file 41598_2021_1033_MOESM5_ESM.docx]

**Title:**

Measurement of Chest Wall Motion Using a Motion Capture System with the One-pitch Phase Analysis Method

**Authors’ full names:**

Hiroyuki Tamiya, M.D., Ph.D. ^1)^, Akihisa Mitani*, M.D., Ph.D. ^1, 2)^, Hideaki Isago, M.D., Ph.D. ^1,3)^, Taro Ishimori, M.D., Ph.D. ^1)^, Minako Saito, M.D., Ph.D. ^1, 2)^, Taisuke Jo, M.D., Ph.D. ^1,2)^, Goh Tanaka, M.D., Ph.D. ^1)^, Shintaro Yanagimoto, M.D., Ph.D. ^4)^, Takahide Nagase, M.D., Ph.D. ^1)^

***Corresponding author**

**Authors’ affiliations:**

^1)^ The Department of Respiratory Medicine, The University of Tokyo Hospital, 7-3-1, Hongo, Bunkyo-ku, Tokyo 113-8655, Japan

^2)^ Health Service Center, The University of Tokyo, 7-3-1 Hongo, Bunkyo-ku, Tokyo, 113-8655, Japan

^3)^ The Department of Clinical Laboratory, The University of Tokyo Hospital, 7-3-1, Hongo, Bunkyo-ku, Tokyo 113-8655, Japan

^4)^ The Division for Health Service Promotion, The University of Tokyo, 7-3-1, Hongo, Bunkyo-ku, Tokyo 113-8655, Japan

**Corresponding author full contact details:**

Akihisa Mitani, M.D., Ph.D

Address: The Department of Respiratory Medicine, The University of Tokyo Hospital, 7-3-1,

Hongo, Bunkyo-ku, Tokyo, 113-8655, Japan

Email: mitania-int@h.u-tokyo.ac.jp

TEL: +81-3-3815-5411

Fax: +81-3-3814-0021

**Table S1. Participants’ characteristics, analyzed based on the presence or the absence of asthma history**

|  | Participants without a history of asthma  (*n* = 37) | Participants with a past history of asthma  (*n* = 11) | *p*-value |
| --- | --- | --- | --- |
| ***Physical examination data*** |  |  |  |
| Age (y) | 19 (3) | 18 (0) | 0.74 |
| Body height (cm) | 170 (6.8) | 170 (4.8) | 0.96 |
| Body weight (kg) | 60 (7.3) | 63 (6.0) | 0.24 |
| Body mass index (kg/m^2^) | 21 (2.5) | 22 (1.9) | 0.09 |
| Arterial oxygen saturation of pulse oximetry (%) | 97 (1) | 97 (1) | 0.24 |
| Pulse rate | 77 (13) | 74 (10) | 0.35 |
| Chest circumference (inspiration) (cm) | 89 (4) | 91 (5) | 0.36 |
| Chest circumference (expiration) (cm) | 84 (4) | 86 (5) | 0.25 |
|  |  |  |  |
| ***Spirometry data*** |  |  |  |
| VC (L) | 4.25 (0.66) | 4.56 (0.49) | 0.13 |
| VC, % of predicted | 94.3 (12.2) | 102 (9.05) | 0.06 |
| TV (L) | 0.79 (0.35) | 0.89 (0.37) | 0.41 |
| FVC (L) | 4.28 (0.80) | 4.55 (0.47) | 0.26 |
| FVC, % of predicted | 94.8 (15.5) | 101 (7.82) | 0.18 |
| FEV1 (L) | 3.96 (0.68) | 4.12 (0.42) | 0.53 |
| FEV1 (L), % of predicted | 98.0 (14.7) | 102 (6.70) | 0.56 |
| FEV1/FVC (%) | 92.9 (5.69) | 90.8 (6.06) | 0.24 |
| FEV1/FVC, % of predicted | 103 (6.20) | 101 (6.82) | 0.22 |
| PEF (L/s) | 8.18 (1.98) | 8.92 (1.89) | 0.30 |
| PEF (L/s), % of predicted | 85.7 (19.8) | 93.8 (19.4) | 0.25 |
| FEF25–75 (L/s) | 5.26 (1.08) | 4.93 (0.93) | 0.48 |
| FEF25–75, % of predicted | 127 (30.4) | 118 (30.1) | 0.45 |
| FEF50 (L/s) | 5.72 (1.21) | 5.40 (1.07) | 0.66 |
| FEF50, % of predicted | 92.1 (18.8) | 86.8 (16.7) | 0.63 |
| FEF75 (L/s) | 3.32 (0.94) | 2.97 (0.83) | 0.25 |
| FEF75, % of predicted | 91.6 (24.6) | 81.8 (21.9) | 0.16 |
| FEF50/FEF75 | 1.81 (0.41) | 1.89 (0.39) | 0.74 |

Data are expressed as mean (SD).

*VC* vital capacity, *TV* tidal volume, *FVC* forced vital capacity, *FEV1* Forced vital capacity, *PEF* peak expiratory flow, *FEF25–75* forced expiratory flow between 25% and 75% of vital capacity, *FEF 50* forced expiratory flow at 50% of forced vital capacity, *FEF75* forced expiratory flow at 25% of forced vital capacity
