# Supplementary material for: Measurement of chest wall motion using a motion capture system with the one-pitch phase analysis method
Source: Sci Rep. 2021 Nov 2;11:21497. doi: 10.1038/s41598-021-01033-8 (PMC8563798; doi:10.1038/s41598-021-01033-8)
Supplement: Supplementary file 6 — Supplementary Table S2. [file 41598_2021_1033_MOESM6_ESM.docx]

**Title:**

Measurement of Chest Wall Motion Using a Motion Capture System with the One-pitch Phase Analysis Method

**Authors’ full names:**

Hiroyuki Tamiya, M.D., Ph.D. ^1)^, Akihisa Mitani*, M.D., Ph.D. ^1, 2)^, Hideaki Isago, M.D., Ph.D. ^1,3)^, Taro Ishimori, M.D., Ph.D. ^1)^, Minako Saito, M.D., Ph.D. ^1, 2)^, Taisuke Jo, M.D., Ph.D. ^1,2)^, Goh Tanaka, M.D., Ph.D. ^1)^, Shintaro Yanagimoto, M.D., Ph.D. ^4)^, Takahide Nagase, M.D., Ph.D. ^1)^

***Corresponding author**

**Authors’ affiliations:**

^1)^ The Department of Respiratory Medicine, The University of Tokyo Hospital, 7-3-1, Hongo, Bunkyo-ku, Tokyo 113-8655, Japan

^2)^ Health Service Center, The University of Tokyo, 7-3-1 Hongo, Bunkyo-ku, Tokyo, 113-8655, Japan

^3)^ The Department of Clinical Laboratory, The University of Tokyo Hospital, 7-3-1, Hongo, Bunkyo-ku, Tokyo 113-8655, Japan

^4)^ The Division for Health Service Promotion, The University of Tokyo, 7-3-1, Hongo, Bunkyo-ku, Tokyo 113-8655, Japan

**Corresponding author full contact details:**

Akihisa Mitani, M.D., Ph.D

Address: The Department of Respiratory Medicine, The University of Tokyo Hospital, 7-3-1,

Hongo, Bunkyo-ku, Tokyo, 113-8655, Japan

Email: mitania-int@h.u-tokyo.ac.jp

TEL: +81-3-3815-5411

Fax: +81-3-3814-0021

**Table S2. Tidal breathing and slow vital capacity mode parameters measured by MCO, analyzed based on the presence or the absence of asthma history**

|  | Participants without a history of asthma  (*n* = 37) | Participants with a past history of asthma  (*n* = 11) | *p*-value |
| --- | --- | --- | --- |
| ***Wall displacement- and time-derived indices*** |  |  |  |
| TADrb (L_MCO_) | 0.17 (0.13 – 0.24) | 0.24 (0.16 – 0.28) | 0.31 |
| TWDrb (rib cage) (L_MCO_) | 0.06 (0.05 – 0.12) | 0.08 (0.04 – 0.13) | 0.97 |
| mTADsl (L_MCO_) | 1.38 (1.11– 1.66) | 1.47 (1.43 – 1.55) | 0.25 |
| Ti (sec) | 1.55 (1.34 – 2.15) | 1.75 (1.48 – 2.15) | 0.42 |
| Te (sec) | 2.08 (1.85 – 2.66) | 2.30 (1.83 – 2.61) | 0.99 |
| Ttot (sec) | 3.75 (3.24 – 4.99) | 3.80 (3.33 – 4.90) | 0.87 |
| I/E ratio | 1.33 (1.23 – 1.50) | 1.32 (1.18 – 1.39) | 0.20 |
| RR (breath/min) | 16 (12 – 19) | 16 (12 – 18) | 0.87 |
| PTIDR | 0.24 (0.20 – 0.28) | 0.26 (0.21 – 0.28) | 0.83 |
| PTEDR | 0.21 (0.18 – 0.24) | 0.23 (0.18 – 0.28) | 0.61 |
| TIDR50 | 0.16 (0.14 – 0.20) | 0.17 (0.12 – 0.20) | 0.71 |
| TEDR50 | 0.13 (0.10 – 0.17) | 0.16 (0.10 – 0.19) | 0.54 |
|  |  |  |  |
| ***Asynchrony indices*** |  |  |  |
| ***Phase angle*** |  |  |  |
| TAA (upper RC–ABD) (degree) | -6.20 (-14.3 – 5.66) | -4.41 (-11.2 – 5.96) | 0.75 |
| TAA (RC–ABD) (degree) | -6.56 (-15.9 – 5.78) | -3.39 (-11.5 – 5.78) | 0.70 |
| HTA (degree) | 2.34 (-0.94 – 3.81) | 1.47 (0.10 – 2.74) | 0.61 |
| RCA (upper–lower) (degree) | 3.97 (1.15 – 7.80) | 3.86 (0.74 – 6.84) | 0.97 |
|  |  |  |  |
| ***Paradox time*** |  |  |  |
| IPT of upper RC (%) | 1.81 (1.43) | 2.14 (1.68) | 0.54 |
| IPT of lower RC (%) | 2.23 (2.57) | 2.18 (1.96) | 0.95 |
| IPT of ABD (%) | 0.24 (0.58) | 1.91 (5.66) | 0.37 |
| EPT of upper RC (%) | 0.74 (0.75) | 0.83 (1.43) | 0.86 |
| EPT of lower RC (%) | 0.48 (0.59) | 0.73 (1.41) | 0.59 |
| EPT of ABD (%) | 0.67 (0.78) | 0.90 (0.83) | 0.42 |
|  |  |  |  |
| ***Compartmental contribution indices*** |  |  |  |
| cRC (area A+B+E+F) (%) | 47.9 (47.1 – 48.7) | 47.9 (47.3 – 49.0) | 0.71 |
| cABD (area C+D+G+H) (%) | 52.1 (51.3 – 52.9) | 52.1 (51.0 – 52.7) | 0.71 |
| cURC right (area A) (%) | 11.5 (11.2 – 11.8) | 11.6 (11.4 – 12.0) | 0.52 |
| cURC left (area E) (%) | 11.5 (11.2 – 11.7) | 11.6 (11.4 – 11.9) | 0.29 |
| cLRC right (area B) (%) | 12.4 (12.2 – 12.6) | 12.3 (12.2 – 12.4) | 0.45 |
| cLRC left (area F) (%) | 12.5 (12.3 – 12.7) | 12.5 (12.4 – 12.6) | 0.92 |
| cHT right (area A+B) (%) | 23.9 (23.6 – 24.4) | 23.8 (23.5 – 24.6) | 0.92 |
| cHT left (area E+F) (%) | 24.0 (23.5 – 24.4) | 24.1 (23.8 – 24.4) | 0.46 |
| cABD right (area C+D) (%) | 25.6 (25.3 – 26.2) | 25.6 (24.9 – 26.0) | 0.57 |
| cABD left (area G+H) (%) | 26.5 (26.1 – 26.9) | 26.4 (26.1 – 27.0) | 0.90 |

Data are expressed as median (interquartile range) unless otherwise specified. For paradox time indices, data are expressed as mean (± SD). L_MCO_ on the figure axes indicates the volume which is estimated from TA wall displacement that can be expressed in L by MCO method.

*MCO* motion capture using one pitch phase analysis, *TADrb* thoraco-abdominal wall displacement measured by restful breathing, *TWDrb* thoracic wall displacement measured by restful breathing, *mTADsl* the maximum amount of thoraco-abdominal wall displacement measured by a slow expiration after the deepest possible inspiration, *Ti* inspiratory time, *Te* expiratory time, *Ttot* total breath time, *I/E* inspiration time/expiration time, *RR* respiratory rate, *PTIDR* peak tidal inspiratory displacement rate, *PTEDR* peak tidal expiratory displacement rate, *TIDR50* tidal inspiratory displacement rate at 50% of TADrb, *TEDR50* tidal expiratory displacement rate at 50% of TADrb, *TAA* thoraco-abdominal asynchrony, *RC* rib cage, *ABD* abdomen, *HTA* hemi-thoracic asynchrony, *RCA* rib cage asynchrony, *IPT* inspiratory paradox time, *EPT* expiratory paradox time, *cRC* compartmental contribution of the rib cage to the total thoraco-abdominal wall movement, *cABD* compartmental contribution of the abdomen to the total thoraco-abdominal wall movem, *cURC* compartmental contribution of the upper rib cage to the total thoraco-abdominal wall movement, *cLRC* compartmental contribution of the lower rib cage to the total thoraco-abdominal wall movement, *cHT* compartmental contribution of the hemithorax to the total thoraco-abdominal wall movement, *cABD* compartmental contribution of the abdomen to the total thoraco-abdominal wall movement
