# Supplementary material for: Measurement of chest wall motion using a motion capture system with the one-pitch phase analysis method
Source: Sci Rep. 2021 Nov 2;11:21497. doi: 10.1038/s41598-021-01033-8 (PMC8563798; doi:10.1038/s41598-021-01033-8)
Supplement: Supplementary file 7 — Supplementary Table S3. [file 41598_2021_1033_MOESM7_ESM.docx]

**Title:**

Measurement of Chest Wall Motion Using a Motion Capture System with the One-pitch Phase Analysis Method

**Authors’ full names:**

Hiroyuki Tamiya, M.D., Ph.D. ^1)^, Akihisa Mitani*, M.D., Ph.D. ^1, 2)^, Hideaki Isago, M.D., Ph.D. ^1,3)^, Taro Ishimori, M.D., Ph.D. ^1)^, Minako Saito, M.D., Ph.D. ^1, 2)^, Taisuke Jo, M.D., Ph.D. ^1,2)^, Goh Tanaka, M.D., Ph.D. ^1)^, Shintaro Yanagimoto, M.D., Ph.D. ^4)^, Takahide Nagase, M.D., Ph.D. ^1)^

***Corresponding author**

**Authors’ affiliations:**

^1)^ The Department of Respiratory Medicine, The University of Tokyo Hospital, 7-3-1, Hongo, Bunkyo-ku, Tokyo 113-8655, Japan

^2)^ Health Service Center, The University of Tokyo, 7-3-1 Hongo, Bunkyo-ku, Tokyo, 113-8655, Japan

^3)^ The Department of Clinical Laboratory, The University of Tokyo Hospital, 7-3-1, Hongo, Bunkyo-ku, Tokyo 113-8655, Japan

^4)^ The Division for Health Service Promotion, The University of Tokyo, 7-3-1, Hongo, Bunkyo-ku, Tokyo 113-8655, Japan

**Corresponding author full contact details:**

Akihisa Mitani, M.D., Ph.D

Address: The Department of Respiratory Medicine, The University of Tokyo Hospital, 7-3-1,

Hongo, Bunkyo-ku, Tokyo, 113-8655, Japan

Email: mitania-int@h.u-tokyo.ac.jp

TEL: +81-3-3815-5411

Fax: +81-3-3814-0021

**Table S3. Forced breathing parameters measured by MCO, analyzed based on the presence or the absence of asthma history**

|  | Participants without a history of asthma  (*n* = 37) | Participants with a past history of asthma  (*n* = 11) | *p*-value |
| --- | --- | --- | --- |
| mTADf (L_MCO_) | 1.31 (1.24 – 1.61) | 1.54 (1.51 – 1.65) | 0.10 |
| fTAD1 (L_MCO_) | 0.83 (0.60 – 1.10) | 0.87 (0.57 – 1.02) | 0.84 |
| fTAD1/mTADf (%) | 60.8 (44.9 –76.8) | 57.7 (36.6 – 71.8) | 0.34 |
| TADpf ^a^ | 1.57 (1.15 – 2.27) | 1.58 (1.29 – 1.96) | 0.83 |
| TADff_50_^a^ | 1.06 (0.67 – 1.53) | 1.02 (0.80 – 1.28) | 0.94 |
| TADff_75_^a^ | 0.40 (0.23 – 0.79) | 0.44 (0.37 – 0.60) | 0.81 |
| TADff_50_/TADff_75_^a^ | 2.19 (1.76 – 3.21) | 2.14 (1.80 – 3.01) | 0.99 |

Data are expressed as median (interquartile range). L_MCO_ on the figure axes indicates the volume which is estimated from TA wall displacement that can be expressed in L by MCO method.

*MCO* motion capture using one pitch phase analysis, *mTADf* the maximum amount of thoraco-abdominal wall displacement measured by a forced expiration after the deepest possible inspiration, *fTAD1* ­the amount of thoraco-abdominal wall displacement in first one second measured by a forced expiration after the deepest possible inspiration, *TADpf* peak expiratory flow derived from flow ­­­­–tho­raco-abdominal wall displacement curve, *TADff_50_* forced expiratory flow rate at 50% of mTADf, *TADff_75_* forced expiratory flow rate at 25% of mTADf

^a^ TADpf, TADff_50_, TADff_75_, and TADff_50_/TADff_75_ were not evaluated due to unstable breathing in four participants (three in participants without asthma history and one in participants with asthma history).
